# At last, a Pennsylvanian stem-stonefly (Plecoptera) discovered

**DOI:** 10.1186/1471-2148-11-248

**Published:** 2011-08-31

**Authors:** Olivier Béthoux, Yingying Cui, Boris Kondratieff, Bill Stark, Dong Ren

**Affiliations:** 1 Key Laboratory of Insect Evolution and Environmental Changes, College of Life Science, Capital Normal University,105 Xisanhuanbeilu, Beijing, 100048, China; 2 40 rue d'Aveillans, La Motte d'Aveillans, 38770, France; 3 Department of Bioagricultural Sciences and Pest Management, Colorado State University, Fort Collins, CO 80523, USA; 4 Department of Biology, Mississippi College, Clinton, MS 39058, USA

## Abstract

**Background:**

Stem-relatives of many winged insect orders have been identified among Pennsylvanian fossils (Carboniferous Period). Owing to their presumed 'basal' position in insect phylogeny, stoneflies were expected to occur at this period. However, no relative has ever been designated convincingly.

**Results:**

In this paper, we report specimens belonging to a new fossil insect species collected from the Tupo Formation (Pennsylvanian; China). The wing venation of *Gulou carpenteri ***gen. et sp. nov**. exhibits character states diagnostic of the order Plecoptera, but lack character states shared by unequivocal representatives of the order. Derived from this identification, the delimitation of the fossil species is ascertained based on comparison of several extant stonefly species. This comparative analysis allowed a trait present in *G. carpenteri ***gen. et sp. nov**., but rarely occurring in extant species, to be documented and highlighted as atavistic. Affinities of taxa formerly proposed as putative stem-stoneflies are reconsidered in the light of the new discovery.

**Conclusions:**

*Gulou carpenteri ***gen. et sp. nov**. is considered the only genuine Plecoptera reported from the Pennsylvanian. Continuing efforts on the systematics of Pennsylvanian winged insects indicate a fauna more diverse than previously appreciated. It suggests that insects already had a long, yet undocumented, history by this time.

## Background

Investigating the early evolution of winged insects (Pterygota) is a thrilling, yet challenging, endeavour. The earlier entomofaunas are documented based on only a few localities of the Pennsylvanian (Carboniferous Period; *c*. 320 Ma [1,2]). These entomofaunas differ substantially from extant ones in the relative abundance of major taxa, among other aspects. The main components are extinct (*e.g*., Palaeodictyopteroidea), and stem relatives of not-so-diverse groups such as Orthoptera (*i.e*. grasshoppers, crickets & wetas), Odonata (*i.e*. dragonflies & damselflies), Grylloblattida (or -odea; *i.e*. ice- or rock-crawlers), and Dictyoptera (cockroaches, termites & mantises, and stem-relatives) [1,3]. In addition, several groups (*e.g*., 'paoliids') are yet of uncertain affinities at the ordinal level [4]. Recently, a stem-Amphiesmenoptera/Antliophora (caddisflies, butterflies & moths/true flies, scorpionflies, fleas) [5] and a stem-Coleoptera (beetles; [6]), were identified. The relative low number of known Pennsylvanian holometabolous insects contrasts sharply with the extant mega-diversity of these groups [1].

Provided the reported occurrences and phylogenetic hypotheses [7,8], stem-Hymenoptera (ants, bees, wasps) and stem-Plecoptera (stoneflies) should occur in Pennsylvanian samples. However, no bona fide fossils have ever been documented.

The oldest entomofauna from China, namely the 'Qilianshan entomofauna', was discovered at the locality of Xiaheyan Village (Zhongwei City, Ningxia Hui Autonomous Region, China). Recent investigations on this Pennsylvanian material recovered the expected Palaeodictyopteroidea [9], stem-Orthoptera [9-11], stem-Odonata [12,13], stem-Grylloblattida [14,15], and stem-Dictyoptera [16,17]. Based on new specimens from this locality, we report a new species, comparatively uncommon and tiny. According to following comparative analysis, it is concluded that this is the earliest stonefly (Plecoptera) yet to be described from the fossil record, and merits assignment to its own new genus and family.

## Results

### Fossil material

**Plecoptera **Burmeister, 1839

**Gulouidae **Béthoux, Cui, Kondratieff, Stark & Ren, **fam. nov**.

**Type genus: ***Gulou *Béthoux, Cui, Kondratieff, Stark & Ren, **gen. nov**.

**Diagnosis: **By monotypy, see that of the type genus.

**Remark: **The type-species of the type-genus, *Gulou carpenteri ***gen. et sp. nov**. is easily distinguished from all known stonefly taxa. In particular, in forewings, no other stonefly taxon lacks the ra-rp specialized cross-vein and possesses a branched MP (and see species-level diagnosis, and Discussion section; [18-21]). Therefore it cannot be assigned to any known family and genus, and the erection of new ones is well-granted. It appears unnecessary to erect taxa of super-familial and infra-ordinal ranks at this time.

***Gulou ***Béthoux, Cui, Kondratieff, Stark & Ren, **gen. nov**.

**Type species: *Gulou carpenteri ***Béthoux, Cui, Kondratieff, Stark & Ren, **gen. et sp. nov**.

**Diagnosis: **By monotypy, see that of the type species.

**Etymology: **From 'gu' and 'lou', 'old' and 'stonefly' in Chinese, respectively.

**Figure 1:**
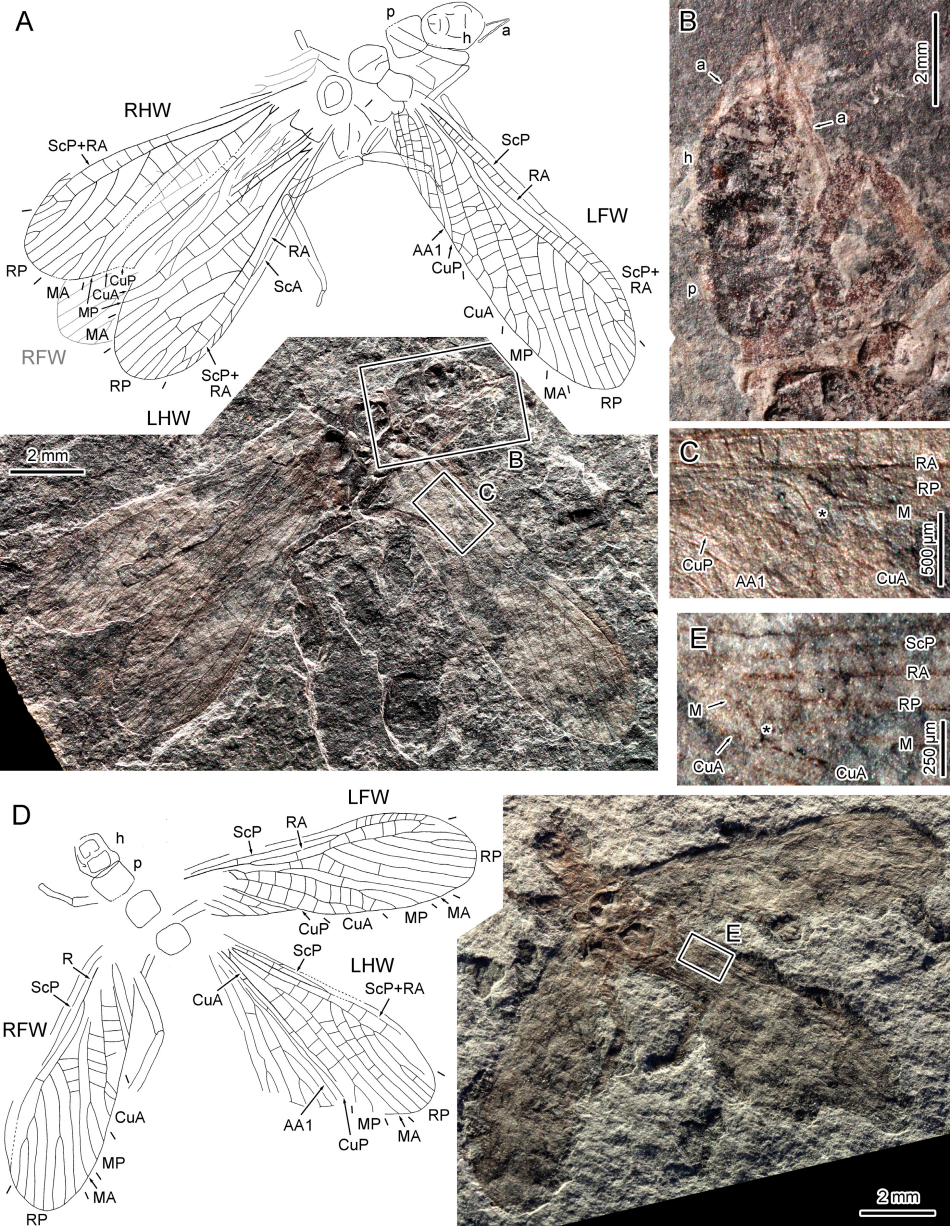
***Gulou carpenteri *gen. et sp. nov. (Pennsylvanian; Xiaheyan Village, Tupo Formation, Ningxia, China)**. A-C, Specimen CNU-NX1-143, holotype; A, drawing and photograph (negative imprint); B, detail of head and fore leg, as located on A (negative imprint); C, detail of forewing arculus (*), as located on A (negative imprint, light-mirrored); D, E, Specimen CNU-NX1-144; D, drawing (RHW omitted) and photograph (positive imprint); E, detail of hind wing arculus (*), as located on D (positive imprint).

**Figure 2:**
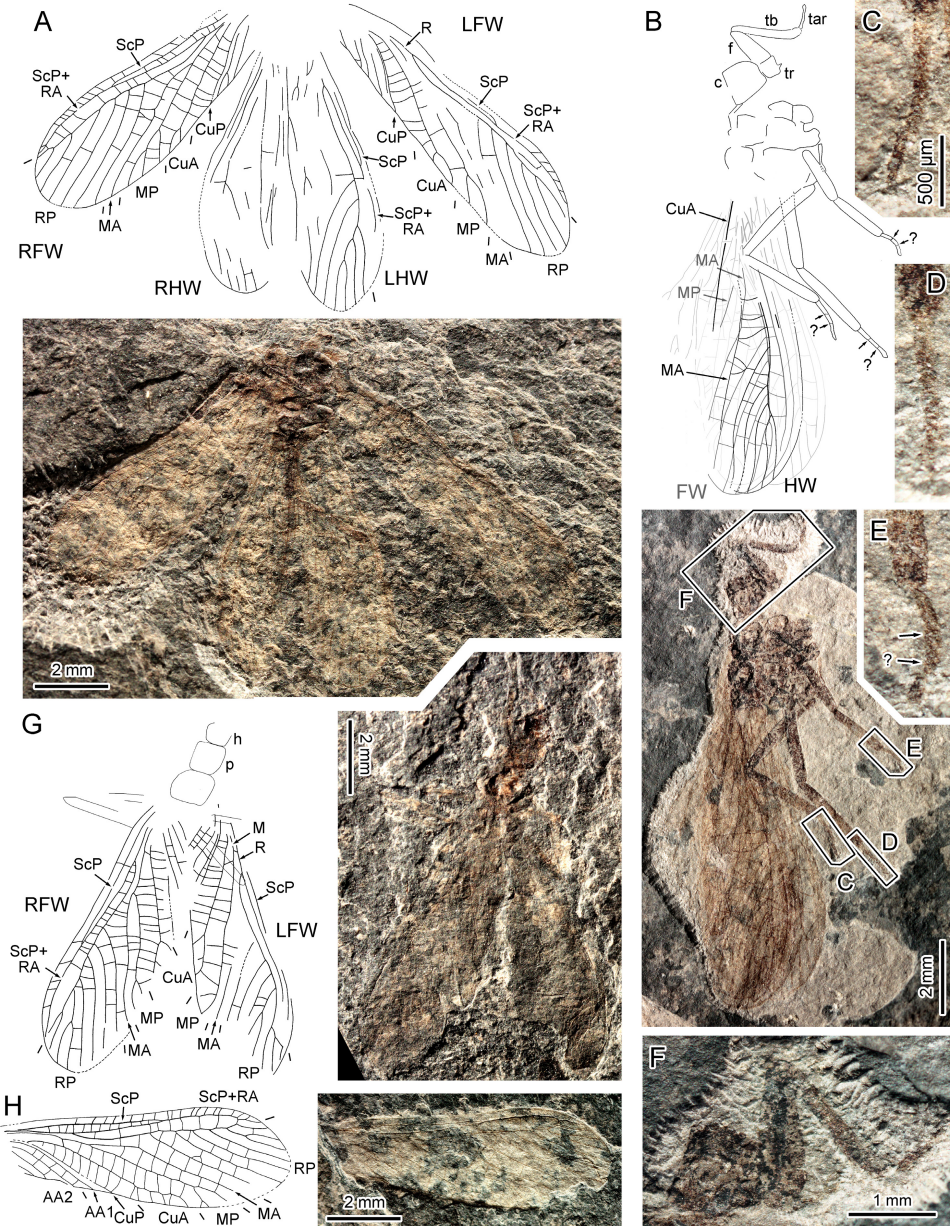
***Gulou carpenteri *gen. et sp. nov. (Pennsylvanian; Xiaheyan Village, Tupo Formation, Ningxia, China)**. A, Specimen CNU-NX1-141, drawing and photograph (negative imprint); B-F, Specimen CNU-NX1-145; B, drawing and photograph (imprint polarity unknown); C, D, detail of hind leg tarsi, as located on B; E, detail of mid leg tarsus, as located on B (with tentative interpretation of segmentation); F, detail of fore leg, as located on B; G, Specimen CNU-NX1-140, drawing and photograph (negative imprint); H, Specimen CNU-NX1-146, drawing and photograph (negative imprint, light-mirrored).

***Gulou carpenteri ***Béthoux, Cui, Kondratieff, Stark & Ren, **gen. et sp. nov**. (Figures 1, 2)

**Diagnosis: **Forewings: ScP reaching RA; RP originating from R at ¼ of wing length; MP and CuA branched distally, with 2-3 distal branches (rarely 4); occurrence of an arculus between M and CuA; cross-veins numerous, unspecialized (except for the arculus). Hind wings: ScP reaching RA; RP originating from R close to wing base, not fused with MA; area between MA and MP narrow; occurrence of an arculus between M and CuA; CuA simple; fold located posterior to/along AA1; vannus well developed; cross-veins numerous, unspecialized (except for the arculus).

**Etymology: **In honor of Prof. F. M. Carpenter, for his important contribution to the study of fossil insects, in particular Palaeozoic fauna.

**Material: **Holotype specimen: CNU-NX1-143; additional specimens: CNU-NX1-137 to CNU-NX1-142, and CNU-NX1-144 to CNU-NX1-159.

**General description: **(measurements probably affected by deformation) body length about 8.9-9.8 mm (based on specimens CNU-NX1-139 and CNU-NX1-142); antenna about 1.5 longer than head (at least); prothorax quadrangular; legs slender; forewings: average length 8.1 mm (min. 5.2 mm, max. 10.5 mm), average width 2.6 mm (min. 1.8 mm, max. 3.4 mm), about twice longer than abdomen; ScP reaching RA just distal to 1/2 wing length; ScP + RA simple; RP originating from R at ¼ of wing length; RP posteriorly pectinate, usually with 6 distal branches (about 54% of observed forewings), rarely with 7 (37%) or 8 (8%); first posterior branch of RP diverging basal to the end of ScP (on RA); MA originating obliquely from M, simple; MP branched, usually distally, rarely at mid-length, with 2 (78%) to 3 (22%) branches; occurrence of an arculus between M and CuA (observed in 15 forewings); CuA branched distally, usually with 3 branches (59%), rarely with 2 (32%) or 4 (9%); areas between MP and CuA and between CuA and CuP broad; claval fold running along CuP; CuP simple and straight; area between CuP and AA1 very narrow; AA1 usually simple, rarely forked; AA2 with more than 4 branches; cross-veins numerous, rarely reticulated; hind wings: length range about 6.5-8.7 mm, width range about 2.0-2.9 mm; ScP reaching RA; RP posteriorly pectinate, with 6-7 branches; MA with 1-3 branches; MP with 1-2 branches; area between RP and MA (basal to the first fork of RP) broad; area between MA and MP narrow; CuA, CuP and AA1 straight and simple; areas between CuA and CuP, and between AA1 and CuP very narrow; fold delimiting remigium and vannus located posterior to/along AA1.

**Specimen description: ***Specimen CNU-NX1-143 *(Holotype; Figure 1A-C): negative imprint of a complete left forewing and fragments of a right forewing, pair of hind wings with anal area partly folded and overlapping, abdomen missing; head moderately well-preserved (Figure 1B), about 3.3 mm long, 2.5 mm wide, in prognathous position; antennae wrapped around head capsule, narrow, longer than head; prothorax about 2.1 mm wide; left forewing 9.5 mm long, 2.9 mm wide; RP with 8 branches; MP with 3 branches (4?); arculus clear (Figure 1C); hind wings preserved length 8.6/8.7 mm (left/right hind wing), 2.8/2.9 mm wide; RP with 7 branches; CuA, CuP, AA1 simple and straight; left hind wing: MA forked; MP simple; right hind wing: MA with 3 branches, MP simple; remigium ending along AA1.

*Specimen CNU-NX1-144 *(Figure 1D, E): negative imprint of complete pair of forewings, well-preserved left hind wing, poorly preserved right hind wing overlapping with right forewing, and head and thoracic elements; forewings 8.3/7.5 mm long (left/right forewing), 2.7 mm wide; RP with 7 branches; MP forked; CuA with 3 branches; left hind wing 7.2 mm long, 2.6 mm wide; RP with 6 preserved branches; MA simple, MP forked distally; occurrence of an arculus between M and CuA (Figure 1E); CuA, CuP and AA1 straight and simple; vannus well developed; cross-veins numerous, unspecialized (except for the arculus).

*Specimen CNU-NX1-141 *(Figure 2A): negative imprint of complete wing pairs (7.5/8.7 mm long, 2.7/2.4 mm wide, in right/left forewing), hind wings poorly preserved (6.5/7.1 mm, 2.0/2.3 mm wide, right/left hind wings), head missing, thorax well preserved but various parts unclear, abdomen poorly preserved; MP with 3 distal branches on right forewing, 2 in left forewing; AA1 forked distally in right forewing; simple in left forewing.

*Specimen CNU-NX1-145 *(Figure 2B-F): single imprint of unknown polarity, with four wings overlapping, four legs well preserved; fore leg (Figure 2B, F) coxa quadrate, 0.93 mm wide and 0.95 mm long, femur short and broad (1.18 mm and long 0.47 mm wide in the middle part), tibia 1.90 mm long and 0.21 mm wide, tarsus preserved length 0.63 mm, 0.06 mm wide; middle leg femur and tibia long and narrow (each about 1.6 mm long), basitarsus 0.33 mm long, 2^nd ^and 3^rd ^tarsomere (if any) not evident, arolium evidenced by stronger sclerotization (Figure 2B, E); hind legs femora and tibiae very long (about 2.7/2.4 mm, respectively); tarsus segmentation not evident, with no more than 4 tarsomeres (Figures 2B-D).

*Specimen CNU-NX1-140 *(Figure 2G): positive and negative imprints of an individual showing forewings (7.4/7.6 mm long, 2.5 mm wide, left/right forewing) and few body remains; prothorax quadrangular; legs slender; RP with 8 branches in right forewing; MP forked and CuA with 2 (3?) branches in both forewings.

*Specimen CNU-NX1-146 *(Figure 2H): negative imprint of well-preserved right forewing, 7.7 mm long, 2.6 mm wide; RP with 7 branches; arculus evident; MA simple; MP forked; CuA with 4 branches.

*Specimen CNU-NX1-147*: positive and negative imprint of complete specimen, four wings overlapping, moderately well preserved head and antennae; head 1.8 mm long, 1.0 mm wide; antennae about 2.6 mm long as preserved (apex possibly missing), very narrow.

### Ordinal assignment

For W. Hennig, "the smaller the number of derived characters common to fossils and recent species, the more doubtful is the assignment of fossils to the stem-group of the Plecoptera" [22]. Indeed ordinal assignment of *G. carpenteri ***gen. et sp. nov**. is not immediately apparent. A discussion of the affinities of *G. carpenteri ***gen. et sp. nov**. with respect to stem-Grylloblattida, then to Plecoptera is presented for clarification. Only a few diagnostic characters have been established for the stem-Grylloblattida (also referred to as Protoperlaria and Paraplecoptera; see below). Among these characters, in forewing, 'CuA divided into two main stems near the wing base, with the posterior stem (CuA2) simple' [23]. This character is absent in *G. carpenteri ***gen. et sp. nov**., in which CuA is distally branched (Figures 1, 2). Another character referred to stem-grylloblattids is the forewing 'arculus' [23], herein interpreted as a strong cross-vein [24]. Such an arculus was observed in 15 specimens, including CNU-NX1-143, 144, 141, 140, 146 (Figures 1A, C, D, 2A, G, H, respectively). However the arculus is also a trait consistently occurring in stonefly forewings [24]. Prothoracic winglet-like lobes have been reported in most Palaeozoic stem-grylloblattids [21], but are absent in *G. carpenteri ***gen. et sp. nov**. (Figures 1A, B, D, 2G). However, the polarity of this character is not evident: (1) if the possession of winglet-like lobes is a symplesiomorphy shared by stem-grylloblattids and a number of other Pennsylvanian lineages such as Palaeodictyopteroidea, its lack in *G. carpenteri ***gen. et sp. nov**. excludes relationships with stem-grylloblattids; (2) if the possession of winglet-like lobes is a secondary, derived condition in stem-grylloblattids [25], its lack in *G. carpenteri ***gen. et sp. nov**. is inconclusive, because the species could equally be interpreted as a very 'basal' stem-Grylloblattida. However there is no evident ground for this option. Instead the presence of an arculus, of ScP reaching RA, of a simple MA, and of a broad MP/CuA and CuA/CuP areas in forewings (Figures 1, 2), clearly indicate affinity with the Plecoptera (ScP reaches the anterior wing margin in some Antarctoperlaria only [18], but according to current phylogenetic schemes [26,27], it must be considered as an apomorphy within Plecoptera). In addition the basal area between M, CuA, and the arculus is free of cross-veins (Figures 1A, 2H), unlike in Grylloblattida possessing an arculus [3], but as in all Plecoptera [3,18]. Hind wings provide additional support to this 'Plecoptera hypothesis'. The occurrence of a hind wing arculus (Figure 1A, D, E) is shared by Plecoptera [18,19] and extant Dictyoptera [24,28], but is absent in Grylloblattida [29]. Although systellognathan Plecoptera possess a branched CuA, representatives of the clade Antarctoperlaria and Euholognatha have this vein simple [18,19], indicating that it is the ancestral condition in Plecoptera, shared with *G. carpenteri ***gen. et sp. nov**. In contrast most Grylloblattida (and Dictyoptera [24,28,30]) have CuA branched. Finally the character 'ScP reaching RA' is a plecopteran feature [18] present in *G. carpenteri ***gen. et sp. nov**. and absent in Grylloblattida [29]. Another character of interest is the number of tarsal segments, known to equate three in Plecoptera ([1,31]). However, the preservation of the available material of *G. carpenteri ***gen. et sp. nov**. does not allow a conclusive statement on this character (Figure 2C-F), although a tarsus with more than four segments is very unlikely. In contrast stem-Grylloblattida possess the plesiomorphic condition, *viz*. a 5-segmented tarsus [32].

Comparison with the few genuine Permian stem-Plecoptera, which are likely to be plesiotypic with respect to their extant relatives, provides additional support to our hypothesis: *Palaeoperla exacta *Sharov, 1961 and *Perlopsis filicornis *Martynov, 1940 [20,21], both known from forewings only, share with *G. carpenteri ***gen. et sp. nov**. a very basal origin of RP, an area between RA and RP narrow for a long distance, and CuA with a few and very distal branches. These similarities strongly suggest that *G. carpenteri *is a stem-Plecoptera. The confirming character states of the above are, in forewing: ScP reaching RA; occurrence of an arculus; area between M and CuA, basal of the arculus, free of cross-veins; MA simple; CuA with a few distal branches; broad MP/CuA and CuA/CuP areas (Figures 1, 2); and in hind wing: ScP reaching RA; occurrence of an arculus; CuA simple (Figure 1A, D).

**Figure 3:**
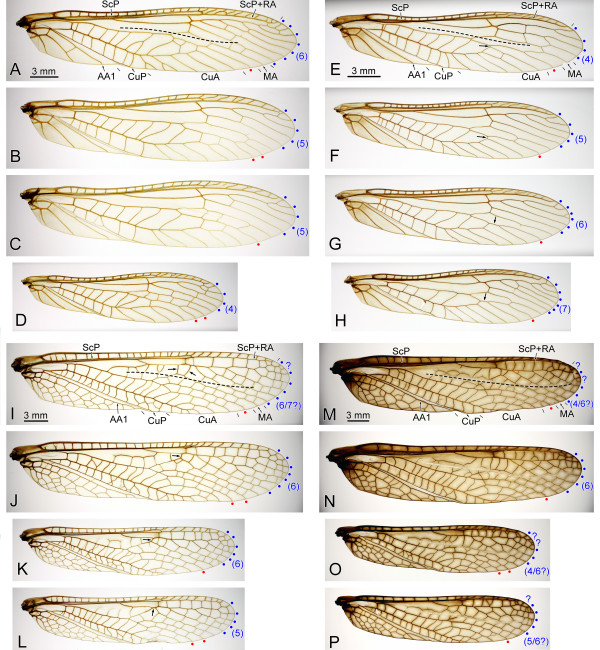
**Selection of extant stonefly forewings for comparison with fossil material**. A-D, *Acroneuria abnormis *Newman, 1838; A, Specimen IWC OB 799 (♀), left forewing; B-C, Specimen IWC OB 800 (♀); B, Left forewing; C, Right forewing; D, Specimen IWC OB 810 (♂), right forewing; E-H, *Acroneuria carolinensis *Banks, 1905 (large arrows indicate various conditions of the connection between MP and CuA); E, Specimen IWC OB 833 (♀), left forewing; F-G, Specimen IWC OB 834 (♀); B, Left forewing; C, Right forewing; H, Specimen IWC OB 838 (♂), left forewing; I-L, *Pteronarcys californica *Newport, 1851 (large arrows indicate various conditions of the connection between RP and MA); I-J, Specimen IWC OB 816 (♀); I, Left forewing; J, Right forewing; K-L, Specimen IWC OB 824 (♂); K, Left forewing; L, Right forewing; M-P, *Pteronarcys princeps *Banks, 1907; M-N, Specimen IWC OB 828; M, Left forewing; N, Right forewing; O-P, Specimen IWC OB 830 (♂); O, Left forewing; P, Right forewing. RP and MP branches indicated by blue and red dots, respectively; r-m fold indicated by a dashed line.

*Gulou carpenteri ***gen. et sp. nov**. exhibits several plesiomorphies with respect to other Plecoptera (fossil or extant), notably its branched MP in forewing (simple in other stoneflies). It must be noticed however that a branched MP was observed as a rare trait in three of the four surveyed extant species (Figure 3). It can be interpreted as an atavism that reinforces the 'Plecoptera hypothesis'. The hind wing of *G. carpenteri ***gen. et sp. nov**. is also plesiomorphic in that RP does not fuse with MA (Figure 1D, E), unlike in other Plecoptera [18]. Finally the abundance of cross-veins in both fore- and hind wing, contrasting with the few generally occurring in stoneflies [18,19,21], and the lack of specialized ra-rp cross-vein, are also plesiomorphic conditions observed in *G. carpenteri ***gen. et sp. nov**. (Figures 1, 2). The combination of character states exhibited by *G. carpenteri ***gen. et sp. nov**. is therefore unique, and supports the establishment of a new family, genus and species.

### *Gulou carpenteri *species delimitation

Fossil specimens listed above are assigned to this single species. The observed variation is limited to wing size and aspect ratio, and traits of wing venation. Size variation falls within the range intra-specific variation as observed in the surveyed extant stoneflies: males are consistently smaller than females (up to 75% of female wing length; Figure 3). In addition differences in size and aspect ratio have been amplified by sediment deformation (obvious in Figure 2A), earlier reported for the locality [10,15,16]. Owing to its size, the holotype specimen CNU-NX1-143 is probably a female. Variation in venation is limited to the number of RP, MP, and CuA branches. The number of RP branches varies from six to eight. Although count of RP branches can be complicated in species of the genus *Pteronarcys *(Pteronarcyidae) by the occurrence of apical 'veinlets' that could either be cross-veins or actual main vein branches (Figure 3I, M, O-P, blue dots and numbers), observation of *Acroneuria *(Perlidae) material provides a conclusive clue (cross-veins are almost completely absent in the apical area; Figure 3A-H): a variation of four to seven branches was observed in the Nearctic *A. abnormis *(Newman) and *A. carolinensis *(Banks), indicating that variation observed among fossil specimens appears limited.

The number of MP branches ranges from two to three among specimens of *G. carpenteri ***gen. et sp. nov**. (Figures 1, 2), but a similar range was observed in three of the surveyed extant stoneflies species (Figure 3, red dots), often at the intra-individual level (Figure 3B-C, 3I-J, K-L, O-P; albeit a two-branched MP is a rare feature in each case). Regardless, the fossil specimen assigned to *G. carpenteri *and reproduced in Figure 2A exhibits a three- and two-branched MP in the right and left forewings, respectively.

Regarding branches of CuA, the range of variation is difficult to determine in *G. carpenteri ***gen. et sp. nov**. because of terminal 'veinlets' that could either be a cross-vein or an actual CuA branch. However, it is mostly restricted to two to three, rarely four, which is minimal in regard of variability observed in other traits, and in extant material.

Only a few specimens of *G. carpenteri ***gen. et sp. nov**. were available for study and it is not unlikely that intra-specific variability in this species is under-estimated. Rare connection of the anterior branch of CuA with MP (variable trait in *A. carolinensis*; arrows on Figure 3E-H), and connection of MA with RP (variable trait in *P. californica*; arrows on Figure 3I-J) could be observed in *G. carpenteri ***gen. et sp. nov**. if more material is discovered.

## Discussion

*Gulou carpenteri ***gen. et sp. nov**. is assigned to a Pennsylvanian stem-Plecoptera. This species is therefore the earliest representative of this lineage, previously documented as early as the Lower Permian [33-35]. It allows a reconsideration of the long-lasting hypothesis of close relationships of Plecoptera with stem-Grylloblattida. Indeed taxon names such as 'Protoperlaria', erected by R. J. Tillyard [36], in reference to the holotype of the type-species of the Permian family Lemmatophoridae, are indicative of these presumed affinities. Eighty years later, a photograph of the very same specimen is reproduced by D. Grimaldi and M. S. Engel [1], who indicate that the corresponding species and some others "were primitive relatives of modern stoneflies". According to the taxon name itself, 'Protoperlaria' is perceived as the stem-group from which emerged Plecoptera and is therefore paraphyletic.

Sharov's 'Paraplecoptera' [37,38] is another taxon name for a similar 'wastebasket group' from which Plecoptera presumably diverged [39]. Basically, J. Kukalová-Peck followed this option [40]. And according to its composition, Storozhenko's 'Grylloblattida' is similar to the Protoperlaria and Paraplecoptera [23,29]. However, apart from superficial similarity and symplesiomorphies, ground for this hypothesis is limited. Literature of the 20^th ^century was aptly summarized by W. Hennig: "Sharov's Paraplecoptera are very probably an 'invalid stem-group' [...]. It is not clear whether they include any species that actually belong to the stem-group of the Plecoptera" [22].

**Figure 4:**
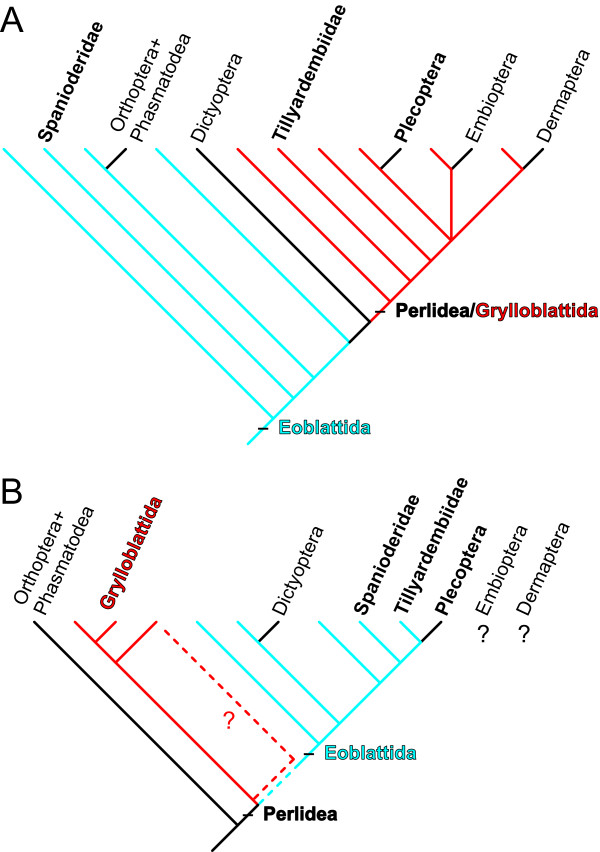
**Recent phylogenetic hypotheses on the position of Plecoptera with respect to fossil taxa (tentative 'cladistic-inspired' representations)**. A, According to various authors [2]; B, According to D. S. Aristov and A. P. Rasnitsyn [25].

Among recent accounts, a paraphyletic Grylloblattida including Plecoptera has been proposed [2]. According to A. P. Rasnitsyn, one of the authors of this collegial contribution, Grylloblattida and Plecoptera, in addition to Dermaptera (earwigs) and Embioptera (webspinners), form a Perlidea superorder [32]. Elsewhere (Figure one in [2]; and Figure 4A), Grylloblattida is represented as paraphyletic, 'giving birth' to Plecoptera, Dermaptera, and Embioptera. It tends to indicate that the taxa 'Grylloblattida' and 'Perlidea' are synonyms then. In the same collegial contribution S. Y. Storozhenko [23] stated that synapomorphies of the order Grylloblattida are "absent because of paraphyletic state of the order in respect to other perlideans [ = stoneflies]". But (1) this author provided no indication about the specific grylloblattid taxon that gave rise to the Plecoptera, and (2) it is unclear how paraphyly can prevent a taxon from possessing apomorphies shared with its 'side-descendants'. Additionally, in the same contribution, A. P. Rasnitsyn dealt with the synapomorphies of Perlidea and stated that "grylloblattideans might take an ancestral state in respect to Gryllidea and so might have no synapomorphies on their own" [32]. In summary 'Grylloblattida' is paraphyletic at an uncertain super-ordinal level and possesses no apomorphy that could have allowed Plecoptera to be assigned to this group.

More recently D. S. Aristov and A. P. Rasnitsyn [25] suggested that the Permian family Tillyardembiidae is sister-group related to Plecoptera, forming a clade whose sister-group is the Pennsylvanian family Spanioderidae. This clade would belong to a paraphyletic order Eoblattida excluding Grylloblattida, both taxa belonging to a large superorder Perlidea (Figure 4B). In other words, Plecoptera would have no direct connection with Grylloblattida, and Eoblattida would no longer include orthopterans.

D. S. Aristov and A. P. Rasnitsyn [25] excluded the Tillyardembiidae from the Grylloblattida, and consider the family to be more closely related to Plecoptera and Spanioderidae instead. The rationale of these authors is not supported due to numerous inconsistencies. For example they stated that "Tillyardembiidae are similar to Spanioderidae in having [...] [a] late forking of M" (p. 260), but their Figure thirteen illustrates a Spanioderidae with "M forks as basal as basal third of wing", a trait stated as limited to Grylloblattida and thus excluding the Tillyardembiidae from this taxon (p. 259). In other words, the Tillyardembiidae are excluded from Grylloblattida and are considered more closely related to Spanioderidae based on this trait absent in the Grylloblattida. The statement "RS [RP] runs only moderately close to R [RA]" is one of "most of the [...] characters [...] shared with Tillyardembiidae [and Spaniodearidae]" is obviously contradicted by "Tillyardembiidae differs from Spanioderidae in having RS [RP] well distant from R [RA]" (p. 260). It is also stated that "Tillyardembiidae are similar to Spanioderidae in having [...] CuA with a backward comb". However this trait occurs in a large number of Grylloblattida [21,29], but also in many 'Eoblattida' other than Spanioderidae, according to the recent account by A. P. Rasnitsyn and D. S. Aristov [41] (p. 13): "The type genus *Eoblatta *Handlirsch, 1906 [...] is the unquestionable centre surrounded with the similar and putatively closely related taxa including *Stenoneura *Brongniart, 1893, *Anegertus *Handlirsch, 1911, [...], and others. All of them share [...] [a] CuA [...] forming a backward comb").

D. S. Aristov and A. P. Rasnitsyn [25] also argue that "M and MP de-sclerotized in vicinity of the fork is known for the Palaeozoic stoneflies" and that it constitutes a similarity between Tillyardembiidae and stoneflies (p. 262). However this 'weak sclerotization' of the stem of M and of MP is present in grylloblattids also [23]. In addition, in stoneflies, this 'desclerotization' is indeed a fold running in the area between R and M, crossing MA (*e.g*. in Pteronarcyidae, Perlidae) or not (*e.g*. in Gripopterygidae), and that can cross branches of RP distally (*e.g*. in Pteronarcyidae); only in a few cases (*e.g*. in Taeniopterygidae) this fold does run close to the stem of M and abut on MA (*i.e*. never approximates MP) [42] (Figure 3). Therefore the 'stonefly r-m fold' and the 'grylloblattid desclerotized M & MP' (present in the Tillyardembiidae) have different locations. In addition a similar fold, located along M (or MP), occurs in many neopteran insects, including Neuroptera (lacewings, owlflies, and antlions) [42]. Therefore this character is not conclusive on relationships of Plecoptera with Tillyardembiidae and Grylloblattida. Unfortunately this character could not be observed on the available material of *G. carpenteri ***gen. et sp. nov**. The only trait that is objectively shared between Spanioderidae (and a number of other stem-Orthoptera), Tillyardembiidae, *G. carpenteri ***gen. et sp. nov**., and Plecoptera is 'ScP reaching RA'. However, several stem-Orthoptera (or 'Eoblattida'), as well as all Palaeozoic and most extant saltatorian Orthoptera, lack this trait [43-47], suggesting that it could have been acquired independently from total-Plecoptera by a lineage of stem-Orthoptera including the Spanioderidae.

Regarding this character, an alternative point of view is provided by A. P. Rasnitsyn and D. S. Aristov [41], in a contribution in which "Structure of the order is Eoblattida discussed [sic]" (p. 13): "*Cheliphlebia *has SC [ScP] entering R [RA] (like in all above [eoblattidan] assemblages, a plesiomorphy after Aristov & Rasnistyn, 2010 [2009])" (original square brackets replaced by brackets). If so the only character shared by the 'assemblage' composed of Spanioderidae, Tillyardembiidae, *G. carpenteri ***gen. et sp. nov**., and Plecoptera would be a plesiomorphy.

Therefore, according to the intricate systematic treatments by D. S. Aristov and A. P. Rasnitsyn [25], and A. P. Rasnitsyn and D. S. Aristov [41], (1) Plecoptera and *G. carpenteri ***gen. et sp. nov**. could equally be a sister-group related to Grylloblattida, the former clade being characterized by the apomorphy 'ScP reaching anterior wing margin', and (2) Spanioderidae and Tillyardembiidae could equally be related to any other 'eoblattid assemblage'.

Finally, the contribution by D. S. Aristov and A. P. Rasnitsyn [25] rests on the debated assumption that a posterior stem of the median system, termed M_5_, occurs in the insect wing venation groundplan. It has been demonstrated that the various 'M_5 _structures' are not homologous [18,24,30]. Assuming close relationships of Spanioderidae with Plecoptera is unfounded, the former family being composed of stem-Orthoptera provided with a common stem M + CuA, a branched CuP, and a fusion of CuA (diverging from M + CuA; *i.e*. the presumed 'M_5_') with a branch of CuP [48]. Occurrence of these traits is not demonstrated for Tillyardembiidae, and they do not occur in Plecoptera (the arculus, *i.e*. the presumed 'M_5_', is a cross-vein) [18].

Notice that Plecoptera be sister-group related to the Tillyardembiidae and the Spanioderidae is not evident from A. P. Rasnitsyn and D. S. Aristov [41]. These authors stated (p. 17) that "Tillyardembiidae [...] is hypothesised to be a dwarf offshoot of [the spanioderid] line, a Permian relict." The Plecoptera (or Perlaria) are not mentioned by these authors [41].

Close relationships of Tillyardembiidae and Spanioderidae with Plecoptera are even more dubious once the wing morphology of *G. carpenteri ***gen. et sp. nov**. and genuine Permian stoneflies is considered. These Palaeozoic Plecoptera have a CuA with a few distal branches [20,21], while CuA is richly branched in Tillyardembiidae and Spanioderidae [25,48]; the Tillyardembiidae do not have the narrow RA/RP areas [25], a trait diagnostic of Palaeozoic (and nearly all) stoneflies [18-21]; set alone the problematic M_5 _/arculus/CuA interpretation, the Spanioderidae do not have the broad MP/CuA and CuA/CuP areas diagnostic of all Plecoptera [18-21]. Provided the antiquity of *G. carpenteri ***gen. et sp. nov**. it is hardly arguable that the Permian Tillyardembiidae constitute a Permian relict of a more basal member of Plecoptera that would have crossed the whole Pennsylvanian without leaving a record. Regarding Spanioderidae, it simply has to be considered as a stem-Orthoptera [48], unrelated to Plecoptera.

In summary we consider that the suggestion of affinities of Spanioderidae with Plecoptera [25] is not grounded. The position of Tillyardembiidae is considered as uncertain. There is no clear synapomorphy supporting the view that Plecoptera actually arose from the known Protoperlaria, Paraplecoptera, stem-Grylloblattida, or 'Eoblattida'. Provided the new record, the argument of 'historical precedence' no longer holds: *G. carpenteri ***gen. et sp nov**. is as old as the oldest stem-Grylloblattida [15]. We conclude that Plecoptera was a distinct lineage as early as the Pennsylvanian, and that known Palaeozoic stem-Grylloblattida might be, at best, remote relatives of Plecoptera.

## Conclusions

Apart from a few Permian genuine Plecoptera [20,21], *G. carpenteri ***gen. et sp. nov**. is the only compelling Palaeozoic stem-Plecoptera, and is the earliest one. Although wing morphology of this species allows some plesiomorphic character states to be outlined for crown-Plecoptera, it provides no conclusive evidence on relationships of this group with other recognized major insect lineages.

Hennig's supposition is supported: "it seems most likely that the Plecoptera arose before the lower Upper Carboniferous" [22]. Indeed continuing identifications of Pennsylvanian stem-relatives of modern groups is beginning to indicate that Pennsylvanian entomofaunas as more diverse than previously appreciated. Many major splits among insect lineages appear to have taken place earlier, during the Mississippian, or even earlier. Unfortunately, no fossil insects are known from this period. As a consequence connecting groundplans of major lineages is difficult to achieve yet. Also, the hypothesis of an early flight-related radiation [49] can hardly be tested, because relative dominance of flight and flight-less species shortly after the time of appearance of the trait are unknown. We concur to the view that prospection of favourable Mississippian (or older) localities must become a priority [50].

## Methods

To comply with regulations of the International Code of Zoological Nomenclature (ICZN), we have deposited paper copies of the above article at the American Museum of Natural History (NYC), the National Museum of Natural History (Washington), the Natural History Museum (London), the Brigham Young University (Provo), and the Colorado State University (Fort Collins), and sent copies to various colleagues.

### Wing venation homologies, and abbreviations

We follow the serial insect wing venation groundplan [51,52]. The corresponding wing venation nomenclature is repeated for convenience: ScP, posterior Subcosta; RA, anterior Radius; RP, posterior Radius; M, Media; MA, anterior Media; MP, posterior Media; Cu, Cubitus; CuA, anterior Cubitus; CuP, posterior Cubitus; AA: anterior analis. The strong and oblique cross-vein occurring between M and CuA near the wing base is referred to as the 'arculus' (indicated as 'arc' on illustrations). Alternative interpretations of the arculus are considered in the Discussion section. On figures, right and left forewings are indicated as RFW and LFW respectively, and right and left hind wings as RHW and LHW; head, prothorax, antennae, coxa, trochanter, femur, tibia and tarsus are referred to as 'h', 'p', 'a', 'c', 'tr', 'f', 'tb' and 'tar'. Width of hind wings was measured opposite the end of CuP.

### Fossil material

All specimens were collected from the Pennsylvanian strata near Xiaheyan village (Zhongwei City, Ningxia Hui Autonomous Region, China). Specimens are housed in the Key Lab of Insect Evolution and Environmental Changes, College of Life Science, Capital Normal University, Beijing, China (CNU; Dong Ren, Curator). All fossils were examined using a Leica MZ12.5 dissecting microscope and illustrated with the aid of a drawing tube. All photographs were taken using a digital camera Canon EOS 450D coupled to a MP-E 65 mm macro lens, and are 'dry-ethanol composites' (*i.e*. they are a combination of photographs of a specimen both dry and immersed in ethanol). All photographs were processed using Adobe Photoshop. Photographs indicated as light-mirrored are the product of an optical effect aiming to revert the polarity of an imprint.

### Extant material

Specimens belonging to recent species were prepared to appreciate intra-specific variability in wing venation. Selected species are *Acroneuria abnormis *(Newman) (12 ♀, 7 ♂), *Acroneuria carolinensis *(Banks) (4 ♀, 5 ♂) (both Perlidae), *Pteronarcys californica *Newport(6 ♀, 5 ♂), and *Pteronarcys princeps *Banks (2 ♀, 2 ♂; 1 ♀ with left wings only; 1 ♂ with apex of RFW damaged) (both Pteronarcyidae). Wings were cut off and mounted in white Euparal medium (Asco Laboratories, Manchester, UK). Photographs were taken using a digital camera Canon EOS 450D coupled with a Canon 50 mm macro lens, and were processed using Abobe Photoshop (including manual dusting off).

## Authors' contributions

OB prepared extant and fossil material, contributed to the preparation of figures, and drafted the manuscript, and is the sole responsible for the content of the Discussion section. YC collected data on fossil material, contributed to the preparation of figures, and helped to draft the manuscript. BK and BS collected and determined extant material, and helped to draft the manuscript. DR supervised palaeontological excavations, designed the project, and secured financial support to YC and OB. All authors read and approved the final manuscript.
